# Reducing healthcare burnout through meditation: benefits and challenges

**DOI:** 10.1080/28324765.2025.2477699

**Published:** 2025-03-19

**Authors:** Varnika Fnu, Divya Rajasekaran, Anamika Pilaniya, Kanishk Aggarwal, Mini Virmani, Aachal Gupta, Rohit Jain

**Affiliations:** a Internal Medicine, Maharishi Markandeshwar Institute of Medical Science Research, Mullana, India; b Internal Medicine, SRM Medical College Hospital and Research Center, Kanchipuram, India; c Internal Medicine, University College of Medical Sciences, New Delhi, India; d Internal Medicine, Dayanand Medical College and Hospital, Ludhiana, India; e Clinical Effectiveness & Quality Improvement, Penn Medicine Princeton Medical Center, New Jersey, USA; f Amity Regional High School, Woodbridge, CT, USA; g Internal Medicine, Penn State Milton S. Hershey Medical Center, Hershey, PA, USA

**Keywords:** Meditation, burnout, stress, healthcare workers, mindfulness, mental health, fatigue

## Abstract

Currently, healthcare professionals face challenges such as excessive stress, mental distress, psychiatric issues, and burnout. Contributors include various personal, professional, and environmental stressors such as prolonged work hours, high workload, patient care responsibility, information overload, and financial concerns. Stress and burnout can cause employee turnover, staffing shortages, medical errors, reduced job satisfaction, and reduced interest in pursuing or continuing healthcare as a career. This paper explores meditation as a possible solution for tackling stress- and burnout-related issues among healthcare professionals. We present findings from various studies that have revealed the physiological effects of meditation and demonstrated its impact on stress reduction, cardiovascular health, immune function, brain structure, pain management, and genetic expression. We provide insights into commonly used meditation tools such as mindfulness-based stress reduction (MSBR) programs, challenges, and solutions for implementing meditation-related programs in mainstream healthcare delivery.

## Introduction

Modern medicine often brings challenges to healthcare workers, leading to excessive stress, mental distress, psychiatric issues, and burnout. Burnout is a psychological syndrome resulting from prolonged occupational stress and depletion of personal coping resources, leading to emotional exhaustion, depersonalization, and a reduced sense of personal accomplishment (Thimmapuram et al., 2017). It is a rampant issue among medical practitioners, including students in the United States, residents, and practicing physicians, affecting nearly half of health practitioners. A 2014 survey revealed that 54.4% of US physicians experienced at least one burnout symptom, an increase from 45.5% in 2011 (Patel et al., 2019). Based on the medical specialty and age group of the physician, researchers analyzed loss of productivity due to lost hours as well as expenses related to listing, training and rehiring a physician and suggested an overall cost of $4.6 billion within the American healthcare system and $7,600 per physician (Han et al., 2019). Additionally, burnout increases the risk of medical errors, as shown by a survey of medical residents in Ireland, which found that 64% of those experiencing burnout symptoms reported making a medical error, compared to only 22% of those without burnout symptoms (O’connor et al., 2017). In a survey of surgeons, 8.9% of the respondents reported making a major medical error in the past 3 months with about 30% reported lapse in judgement due to burnout being a major cause (Shanafelt et al., 2010). Burnout also leads to decreased job satisfaction, higher turnover, absenteeism, and strained relationships (Siedsma & Emlet, 2015). Various factors have been shown to contribute to burnout in physicians, such as situational stressors (work hours, sleep deprivation, and staff conflicts), personal stressors (conflicts with family or friends, financial difficulties, or relocating to a new city), and professional stressors (patient responsibilities, teaching or supervisory responsibilities, and information overload, among others) affecting patient outcomes, doctors, and the overall system (Busireddy et al., 2017; Grover et al., 2018)).

Various strategies that have been employed to address burnout include retreats, practicing self-care, spending time with family and limiting work hours. In addition, research has shown that meditation interventions effectively minimizes stress levels and boosts one’s health (Thimmapuram et al., 2019). Meditation, an ancient practice with roots in various spiritual traditions, has emerged as a powerful tool for promoting mental well-being.

*Meditation* encompasses a variety of practices and approaches designed to improve consciousness, cultivate mental clarity, and improve overall psychosocial and physical health. Despite many meditation practices across various cultures, the commonly employed meditation techniques in healthcare settings include mindfulness meditation, Transcendental meditation (TM), loving-kindness meditation (LKM) and yoga, each offering unique benefits such as stress reduction, emotional regulation, and enhanced concentration. By engaging in meditation regularly, individuals can cultivate a greater sense of balance in their lives and achieve a more profound connection to themselves and their surroundings.

Mindfulness, defined as paying attention in a particular way: on purpose, in the present moment, and nonjudgmentally (Black, 2011), is one of the commonly used interventions and includes Mindfulness-based stress reduction (MBSR) and mindfulness cognitive therapy (MBCT). TM is a meditation technique wherein practitioners silently repeat a singular mantra (a sound devoid of meaning) without focus or reflection. LKM is a form of meditation from ancient Buddhist culture (Zeng et al., 2015) in which practitioners focus on kindness to themselves, loved ones, acquaintances, strangers, and all beings. In LKM, practitioners repeat phrases such as “may you be happy” or “may you be free from suffering” toward targets.

There is an abundance of data showing the impact of meditation on healthcare providers (Cohen et al., 2023). Research has indicated that there is a diminishing of stress, anxiety, and burnout syndrome in healthcare practitioners who engage in daily meditations (Krasner et al., 2009). Meditation supports healthcare personnel physically through relaxation and psychologically by creating a protective shield against outside stress and maintaining empathy and compassion for patients. In addition to psychological benefits, meditation often seems to lead to a decrease in blood pressure, enhancement of immunological response, and an overall decrease in inflammation (Black et al., 2015). Various hospitals and healthcare institutions have implemented meditation in their day-to-day work, and numerous studies have proven its efficacy. For instance, the Mayo Clinic in Rochester, Minnesota, began providing mindfulness meditation programs to its workers, reducing burnout and increasing job satisfaction among employees (Amutio et al., 2018). Likewise, the Cleveland Clinic has implemented meditation programs to help decrease stress among the medical personnel who treat patients and enhance patients’ well-being (Shanafelt et al., 2019). Programs such as MBSR and tailored meditation workshops for healthcare professionals show promise in overcoming these obstacles. Allowing meditation to become a keystone for supporting corporate well-being and developing individuals’ psychological defenses can lead to the formation of a healthier, happier, and more productive team.

## Aim

This narrative review aims to bridge knowledge gaps and update practitioners about prevalence and impact of burnout syndrome. In addition, the review will discuss physiological and clinical implications of commonly employed meditation interventions there by offering practical insights for the organizations.

## Methods

This narrative review summarizes the findings of peer-reviewed research published in English from 1990 to 2025. We searched electronic databases, including PubMed, Scopus, Web of Science, and Google Scholar, for pertinent information. The search terms encompassed combinations of keywords related to the following: outcome measures (”burnout‘, ’stress‘, ’well-being‘, ’attention‘, ’empathy‘, ’patient care‘), healthcare provider demographics (’healthcare workers‘, ’physicians‘, ’nurses‘, ’ ‘psychologists’ psychiatrist‘), and meditation methodologies (’meditation‘, ’mindfulness‘, ’Transcendental Meditation”).

The inclusion criteria comprised: (1) studies focused solely on healthcare professionals; (2) meditation-based interventions; (3) assessments of mental health, cognitive function, professional performance, or physiological outcomes; and (4) study designs including observational studies, meta-analyses, systematic reviews, or randomized controlled trials. Studies that exclusively examined non-healthcare populations lacked specific intervention methods, did not provide complete articles, and were produced in languages other than English were rejected. We endeavored to identify common patterns of effects and the degree of evidence for various outcomes while acknowledging the diversity of meditation therapies and outcome measures among studies. As the present study is a narrative review, does not include human participants directly, and utilizes publically accessible sources, institutional review board approval was not necessary.

## Results

### Burn out as a syndrome in health care setting

Healthcare systems should be aware of the elevated rates of occupational burnout. Healthcare providers experience work-related stress, which can result in anxiety, depression, burnout, and psychosomatic disorders, resulting in a decrease in quality of life (Firth-Cozens & Greenhalgh, 1997). ICD 11 defines burnout as a syndrome caused by unmanaged chronic occupational stress, which can lead to tiredness, mental remoteness from work, negativity, cynicism, and decreased professional efficacy. It arises from a mismatch between employment intentions and reality and, over time, may go unnoticed by the affected individual. Yang et al noted increased risk of mood disorders (anxiety, depression and physical illness (flu-like symptoms, insomnia and neck pain) (Yang & Hayes, 2020).

Self-perpetuating burnout often results from inadequate coping strategies with a significant economic impact of such conditions, as shown by data on absenteeism and turnover (Jacobson et al., 1996). Healthcare workers face factors such as increased administrative workload, patient suffering, verbal and physical abuse, workplace bullying, emotional repression, litigation risk, role conflicts, and organizational changes that can lead to distress and burnout (Michie & Williams, 2003). The COVID-19 pandemic has introduced additional stressors to individuals beyond their work responsibilities, such as longer work hours, alterations in childcare and eldercare obligations, ethical dilemmas related to providing care such as rationing, and a certain level of existential questioning, potentially compromising their overall well-being due to underlying or new physical, mental, and social limitations.

Between February 2019 and December 2021, almost 20,627 healthcare providers participated in a survey of approximately 120 healthcare systems. The survey used the Mini Z work-life measure and found burnout affecting over 60% of individuals, with over 40% expressing a desire to quit by 2021 (Linzer et al., 2022). In a separate study involving 20,947 healthcare workers during the COVID-19 pandemic, respondents from 42 companies, 38% reported experiencing anxiety or despair, 43% experienced job overload, and 49% indicated burnout (Prasad et al., 2021). A study evaluating 460 mental health service providers (i.e., social workers, psychologists, and case managers with college, associate, or high school degrees presents a troubling picture regarding the levels of emotional exhaustion (EE) and other adverse work outcomes among American mental health workers (Acker, 2012). More than half of the workers (56%) reported moderate-to-high levels of EE, 73% reported moderate-to-high levels of stress, and half (50%) considered quitting their jobs. A recent meta analysis noted the prevalence of EE around 40% (O’Connor et al., 2018).

The WHO on Health Workforce (WHO, 2020) indicates that an additional 18 million health workers are needed to achieve comprehensive and efficient delivery of health services that are essential for ensuring the well-being of all individuals. It is crucial to prioritize these professionals’ mental well-being to maintain the quality of patient care, particularly considering the increasing demands of caring for aging populations. Workload management is a crucial factor affecting healthcare professionals’ well-being in the health industry. The combination of being subjected to excessive job demands and having no control over one’s workload leads to a significantly elevated degree of stress and burnout. A strong correlation was observed between the ratio of patients to nurses and the occurrence of urinary tract infection (0.86; *p* = .02) and surgical site infection (0.93; *p* = .04). In a multivariate model that accounted for patient severity and nurse and hospital characteristics, only nursing burnout was significantly associated with increased risk of urinary tract infections (0.82; *p* = .03) and surgical site infections (1.56; *p* < .01). Hospitals that experienced a 30% reduction in burnout witnessed a significant decrease of 6,239 infections, resulting in annual cost savings of up to $68 million (Cimiotti et al., 2012). Mitigating burnout among healthcare workers is a viable approach to effectively delivering healthcare.

### Correlation of meditation practice with physiological health

Many studies have revealed the physiological effects of meditation, demonstrating its impact on stress reduction, cardiovascular health, immune function, brain structure, pain management, and genetic expression. A study (Chandran et al., 2021) by researchers at the University of Florida found that eight days of intense meditation caused robust immune system activation. Inner Engineering practices involving simple yoga and meditative practices showed increased activity of 220 genes related to the immune response, including 68 genes associated with interferon signaling, a key part of the body’s defense against viruses and cancer. Mindfulness meditation has been shown to increase the number of CD4+ T helper cells, which play a crucial role in coordinating immune responses. This is particularly beneficial for individuals who are diagnosed with acquired immunodeficiency syndrome (AIDS) (Black & Slavich, 2016). Research by Richard (Davidson et al., 2003). participants in an eight-week mindfulness program demonstrated increased antibody production after receiving a flu vaccine, indicating an enhanced immune response. A meta-analysis of the effects of mindfulness meditation on immune function revealed that meditation is associated with increased activity of natural killer cells and a decrease in markers of inflammation, such as C-reactive protein (CRP). These findings indicate that meditation positively affects the maintenance of the body’s immune defense mechanisms, potentially through stress reduction and improved overall well-being (de Punder et al., 2019). Multiple studies have found that mindfulness meditation reduces the pro-inflammatory signaling pathway NF-kB activity and lowers circulating C-reactive protein (CRP) levels, an inflammatory marker. These reductions hold significant value because chronic inflammation is a precursor to various diseases (Black & Slavich, 2016). Mindfulness meditation has been associated with increased telomerase activity, which helps in protecting against cellular aging. This increase in telomerase activity is important for reversing the adverse effects of immune system aging (immunosenescence) (Davidson et al., 2003; de Punder et al., 2019). Meditation is believed to enhance vagal tone through the cholinergic anti-inflammatory pathway, thereby suppressing the activity of pro-inflammatory cytokines and decreasing overall inflammation in the body. Meditation has also been shown to reduce stress-induced production of interleukin-6 (IL-6), a pro-inflammatory cytokine. This reduction is particularly beneficial for individuals with chronic stress and depression, as elevated IL-6 levels are common in these conditions (Pavlov & Tracey, 2012).

Meditation has been associated with improvements in cardiovascular health as well. Regular practice of static meditation has been linked to decreased levels of triglycerides and, to a lesser extent, total cholesterol levels. This study suggested meditation as a noninvasive supplement to conventional therapies, offering a potentially effective way to improve cardiovascular health (Antonelli et al., 2024). Another study (Loucks et al., 2015) examined the effects of meditation on cardiovascular risk factors among middle-aged adults. Participants who engaged in a 12-week meditation program showed significant reductions in systolic and diastolic blood pressures and improvements in heart rate variability, a marker of autonomic nervous system balance. These findings support the idea that meditation can induce relaxation and counteract sympathetic nervous system overactivity. Meditation is widely recognized for its effectiveness in reducing stress and anxiety.

Researchers have observed a dramatic decrease in stress markers such as cortisol and inflammatory cytokines, suggesting that meditation can alter the stress response system (Hoge et al., 2018). Similar findings were reported by a different study indicating that mindfulness meditation effectively lowers perceived stress and improves emotional regulation (Khoury et al., 2015). Research has indicated that meditation can correct hormonal imbalances. Turakitwanakan et al. (2013) explored the effects of mindfulness meditation on hormone levels in healthy adults. The findings revealed that meditation significantly reduced the levels of cortisol, the primary stress hormone, and increased melatonin levels, which regulate the sleep-wake cycle. These hormone adaptations likely improve meditation’s comprehensive stress-reducing and health-enhancing effects. Meditation boosts neuroplasticity and the brain’s potential for remodeling by generating new neural connections. MRI scans were used in a study that revealed that individuals who practice long-term meditation had increased gray matter density in brain regions associated with learning, memory, and emotional regulation, such as the hippocampus and the prefrontal cortex.

These structural changes suggest that meditation can enhance cognitive functions, including attention, memory, and executive function (Lazar et al., 2005). Meditation also plays a significant role in pain management. Zeidan et al. (2012) demonstrated that mindfulness meditation reduced pain perception and pain-related brain activity. Mindfulness meditation participants reported lower pain intensity and unpleasantness, correlating with decreased activity in the primary somatosensory cortex and increased activation in brain regions associated with cognitive control and emotion regulation. Various studies have shown that meditation also impacts the gut-brain axis and microbiome. Meditation was associated with increased microbial diversity and abundance of beneficial bacteria, such as Lactobacillus and Bifidobacterium. These changes in the gut microbiome correlated with improvements in mood and reductions in anxiety symptoms, suggesting a potential mechanism for the mental health benefits of meditation (Jia et al., 2020).

**Figure 1.**
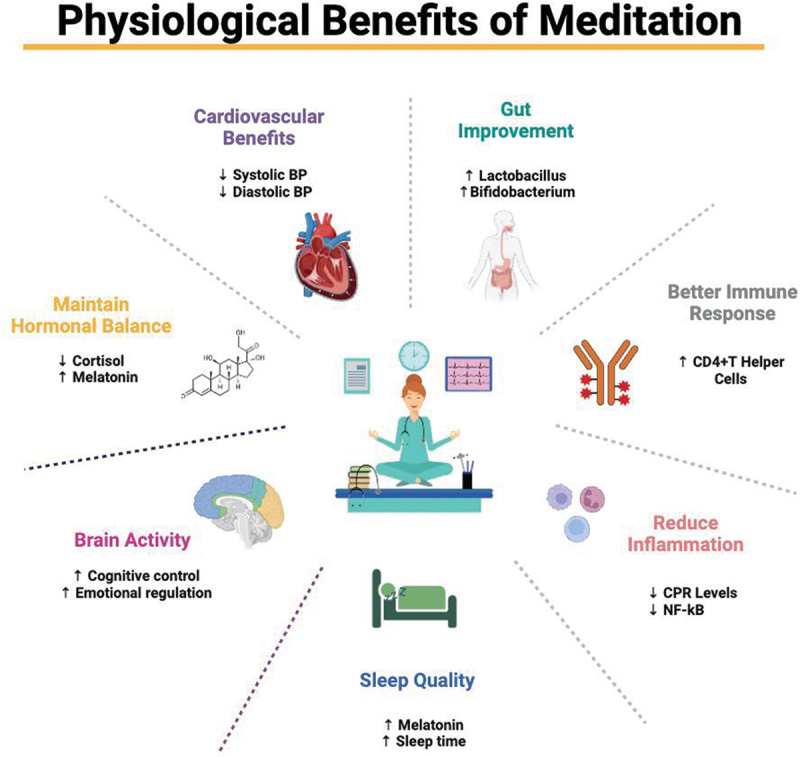
Physiological benefits of meditation.

Meditation has been shown to improve sleep quality. Ong et al. conducted a randomized controlled trial to examine the effects of mindfulness meditation on sleep disturbance. Participants who practiced meditation reported significant improvements in sleep quality, including reduced sleep latency and increased total sleep duration. This study also found reductions in markers of physiological arousal, such as heart rate and sympathetic nervous system activity, which are often elevated in individuals with sleep disorders (Ong et al., 2014). In summary, the physiological effects of meditation are diverse and profound, impacting various aspects of health and well-being. (Figure 1) Meditation offers a holistic approach to enhancing physical and mental health through stress reduction, improved cardiovascular health, enhanced immune function, neuroplasticity, pain management, epigenetic modifications, gut-brain axis modulation, improved sleep quality, and hormonal balance.

## Meditation interventions in healthcare

MSBR is a psychoeducational approach designed by Kabat-Zinn in 1979. The program has a duration of eight weeks and consists of weekly lessons that are 2.5 hours long. Furthermore, there is a specifically assigned “day of silence” that occurs between the sixth and seventh weeks. Participants were taught several meditation techniques that they practiced in class and in daily life. Studies using this program in healthcare professionals have reported reduced stress and emotional fatigue, improved life satisfaction and self-compassion, and enhanced mood (Galantino et al., 2005; Shapiro et al., 2005). In a study examining the impact of a 12-week program called ‘Heartfulness Meditation, individuals who practiced meditation showed considerable improvement in all aspects of burnout and almost all characteristics of emotional well-being. One hundred forty-eight medical, surgical, and primary care residents participated in an eight-week MBSR program. The therapy involved attending 2.5-hour weekly sessions and one full day of silent meditation lasting 6 hours. There was no discernible disparity in emotional fatigue between the two groups. However, the group that received the intervention reported significantly more significant increases in personal accomplishments, worry, mindfulness abilities, self-compassion, and perspective-taking (empathy) (Verweij et al., 2018).

Bosch et al 2024 investigating mindfulness meditation over four weeks among nursing participants revealed that the intervention group felt more competent in addressing challenges and adopted a more balanced perspective on life situations compared to the control group. A separate study involving nurses, allied healthcare professionals, and physicians indicated that a 6-week trainer-guided virtual Heartfulness meditation program resulted in a notable enhancement in workplace burnout and vigor, alongside a trend towards increased compassion satisfaction when compared to gratitude practices. The stress levels dramatically diminished after one week of five-minute mindfulness meditation exercises among 61 busy mental healthcare providers. Participants engaged in seven days of five-minute mindfulness meditation exercises online, intended to assess the efficacy of brief mindfulness practices in diminishing perceived stress levels and enhancing mindfulness, characterized by heightened attentiveness to the present moment in a purposeful and non-judgmental manner (Lam et al., 2015).

Recent meta analyses provide evidence that mindfulness-based interventions can improve the well-being of healthcare professionals sooner (Ong et al., 2024), associated with meaningful reductions in burnout, resulting in lower scores for emotional exhaustion and depersonalization and higher scores for personal accomplishment (Fendel et al., 2021; Suleiman-Martos et al., 2020).

**Transcendental Meditation** among healthcare professionals resulted in substantial decreases in chronic stress in a study conducted at Duke University (Joshi et al., 2022), and in a small cohort of emergency department physicians exhibited notable reductions in burnout, as well as symptoms of depression, anxiety, stress, and sleep disturbances (Azizoddin et al., 2021). TM technique seemingly expedites and significantly alleviates symptoms of healthcare professional burnout, insomnia, and psychological distress, while concurrently enhancing overall well-being. Notably, these substantial impacts were shown as early as two weeks post-test for all outcome measures, except for depersonalization and professional success. At three months post-baseline, the TM group exhibited significant reductions (Nestor et al., 2023).

In another study (Loiselle et al., 2023), TM group at 4 months showed significant improvements in total burnout (*p* = .020) as compared to controls, including the Maslach Burnout Inventory dimensions of despair (*p* = .016), personal accomplishment (*p* = .018), and emotional exhaustion (*p* = .042). In baseline interviews, doctors reported the typical symptoms of despair and burnout. People who used the TM technique on a regular basis reported feeling less of those symptoms. Similar alterations were not reported by the control group. More studies found that the average reduction in symptoms was 62% for anxiety, 58% for somatization, 50% for depression, 44% for insomnia, 40% for emotional exhaustion, 42% for depersonalization, and 18% for well-being (all *p* < 0.004) at 3 months (Nestor et al., 2023).

Chen et al. showed LKM improved physicians’ empathy and communication (Chen 2021). A 10-minute LKM practice increases feelings of social connection and decreases self focus and thus could be a tool that could be applied with minimal time requirements (Seppala et al., 2014). Despite the small sample size, a recent study on Tele-yoga showed the burnout index, PSQI, anxiety, and stress scores, and IL-6 and serum cortisol levels were significantly lower among the tele-yoga group compared to the control group participants (*p* < 0.05) (Naveen et al., 2024).

## Challenges & barriers

Some of the potential barriers (Table 1) for implementation include time constraints, organizational culture and access to resources. One meta-analysis found that the dropout rate from Mindfulness-Based Interventions (MBIs) among individuals diagnosed with anxiety or depressive disorders ranged from 8% to 37%, with a median dropout rate of 15.5% (Strauss et al., 2014). Many individuals find it challenging to allocate time to engaging in daily mindfulness practice. The problems associated with propagating MBIs include a shortage of qualified mindfulness teachers, the financial burden on organizations that are hesitant to participate in group interventions, and the practical difficulties of scheduling courses to accommodate work needs (Wyatt et al., 2014). Some hindrances were long duration, becoming self-critical, and emerging negative thoughts. In contrast, prior knowledge, positive predisposition, motivation to reduce stress, increased self-compassion, and belief in rationale were noted to be facilitators of interventions (Banerjee et al., 2017).

**Table 1.** Comparing challenges and plausible solutions for stress in the healthcare environment

Challenges	Solutions
High Workload and long hours	Adaptable scheduling and sufficient staffing
Emotional stress from patient care	Providing access to mindful meditation programs
Burnout	Recommend taking regular breaks and promoting work life balance
Lack of resources and support	Increased allocation of resources and acquiring additional support staff
Psychological safety	Building a culture of safety and continuous learning

Educating initiatives, technology-based solutions, leadership support, brief mediation format can help facilitate implementation despite the challenges. A study employed Synctuition, an innovative audio mindful meditation software, to take participants on a 20- to 30-minute 3D audio trip to relaxation over 30 days using binaural beats technology and relaxing sounds and frequencies. Using Cohen’s Perceived Stress Scale (Cohen et al., 1994) (ranging from 0–40), participants in general reported improvement in stress (Prado et al., 2023). As part of their Hello Human Compassion campaign, Dignity Health introduced “The Reflective Pause” initiative, encouraging employees to take time each day for quiet contemplation to benefit themselves and build meaningful relationships with peers and patients. Dignity Health recommends taking two minutes daily to “check in” with yourself, whether in the morning, during a work break, during a difficult time or in the evening. People are encouraged to ponder their relationships, employment, and daily life. Participants can share their daily mindfulness practice using #Take2Mins on social media (Minda, 2018).

## Discussion

The evidence as highlighted above suggests that meditation offers substantial benefits for healthcare providers across multiple domains. The strongest evidence supports meditation’s effects on stress reduction, burnout prevention, emotional regulation, attention enhancement, and sleep improvement. Emerging evidence indicates potential benefits for empathy, immune function, cardiovascular health, and possibly error reduction, though these areas require further investigation. MSBR techniques have been employed with success across multiple setups and duration have been modified to facilitate ease of implementation. TM has demonstrated particular efficacy for burnout reduction, as evidenced by the 2022 Duke University randomized controlled trial. This finding holds special relevance given the high prevalence of burnout among healthcare providers and its consequences for both provider well-being and patient care. The relatively straightforward nature of TM practice—20 minutes twice daily of silent mantra meditation—offers practical advantages for implementation in healthcare settings.

With time, more web-based, tele, and mobile applications are being explored to improve ease of use, practicality, and compliance. A web-based training program that encompassed 15-minute exercises per day, 6 days a week, for 6 weeks improved burnout symptoms in practicing psychologists. Mobile meditation applications can be a promising approach to expanding access, as demonstrated by the 2023 study using the Synctuition application.

Despite growing evidence supporting meditation’s benefits for healthcare providers, several limitations in the current research warrant consideration. Because of the variability of the nature of the meditation techniques, methodological heterogeneity such as intervention duration and outcome measures varied between various studies. Selection bias with healthcare providers being represented within certain demographics and institutions can represent overrepresentation and reduce generalizability. Studies lacked long-term follow-up, so sustained outcomes could not be assessed. Certain studies might have been overlooked because of the narrative nature of the review. The studies were included without assessing their methodological rigor, and there is no statistical summary between the studies.

Future research should explore assessing studies with practical and cost-effective interventions, objective biomarkers, healthcare staff outcome studies, and more technological tools, including artificial intelligence applications. Healthcare providers and organizations should continue to implement evidence-based approaches to incorporate mediation practices to improve stress management. Organization leaders and policymakers should prioritize mental health well-being to address burnout. Policy makers can aid in other aspects such as value-based payment models to help alleviate the stress of seeing as many patients as quickly as possible, leveraging social workers and case managers to screen and address social barriers for health care and reducing the burden of clerical works on health care workers (Khullar, 2023). More programs could be included in medical education, as well as continued certification requirements to increase awareness among practitioners.

## Conclusion

Modern medicine often brings challenges to healthcare workers, leading to excessive stress, mental distress, psychiatric issues, and burnout. Mitigating burnout among healthcare workers is a viable approach to effectively delivering healthcare. Research has indicated that there is a decrease in stress, anxiety, and burnout syndrome in healthcare practitioners who engage in daily meditation. Meditation physically and psychologically supports healthcare personnel through relaxation by creating a protective shield against outside stress and maintaining empathy and compassion for patients. In addition to psychological benefits, meditation often leads to a decrease in blood pressure, enhancement of immunological responses, and an overall decrease in inflammation. Various hospitals and healthcare institutions have implemented meditation daily, and numerous studies have proven its efficacy.

However, implementing meditation in care delivery settings is not without its challenges, especially selection bias and time requirement amidst busy schedules. Enhanced organizational focus is needed to integrate meditation into mainstream healthcare delivery and achieve desired psychological outcomes.
